# Physiological effects of autotoxicity due to DHAP stress on *Picea schrenkiana* regeneration

**DOI:** 10.1371/journal.pone.0177047

**Published:** 2017-05-08

**Authors:** Li Yang, Xiao Ruan, Dean Jiang, Jianhong Zhang, Cunde Pan, Qiang Wang

**Affiliations:** 1 Ningbo Institute of Technology, Zhejiang University, Ningbo, China; 2 College of Life Sciences, Zhejiang University, Hangzhou, China; 3 Ningbo Academy of Agricultural Sciences, Ningbo, China; 4 College of Forestry and Horticulture, Xinjiang Agricultural University, Urumqi, China; Murdoch University, AUSTRALIA

## Abstract

*Picea Schrenkiana* as one of the most important zonal vegetation was an endemic species in Middle Asia. Natural regeneration of *P*. *Schrenkiana* is a long existing problem troubling scientists. The autotoxicity of 3,4-dihydroxy-acetophenone (DHAP) was found to be a causative factor causing the failure of *P*. *Schrenkiana* natural regeneration. The effects of concentrations of DHAP treatment on the viability of root cell, activities of antioxidant enzymes and levels of *P*. *Schrenkiana* phytohormones were performed to disclose the physiological mechanism of DHAP autotoxicity. It was observed that high concentration of DHAP could inhibit the seed germination and seedling growth, but had a hormesis at low concentrations. Analyses showed that the root cells significantly lost their viability treated with high DHAP. The enzymes activities of seedlings were significantly stimulated by the treatment of 0.5 mM DHAP to give a transient increase and then decrease as DHAP concentration increased to 1.0 mM except for GR (glutathione reductase) in which DHAP treatment had little effect on its activity. Comparing with the control, an increase in the levels of phytohormones ZT (zeatin), GA_3_ (gibberellic acid) and IAA (indole acetic acid) was induced by the treatment of DHAP at low concentrations (0.1–0.25 mM), but the significant deficiency was found treated by high concentrations (0.5–1.0 mM). In addition, the ABA (abscisic acid) level increased in all experimental observations. These results suggested that DHAP significantly affected indices of growth and physiology, and provided some new information about different effect in *P*. *Schrenkiana* treated with DHAP.

## Introduction

Plant recruitment plays a central role in plant population and dynamic communities [1]. Plant recruitment can be influenced by several parameters including light, nutrients, water, understory vegetation or predation [2–4], and also by the chemically mediated interferences (allelopathy) [5]. Higher plants generally release one or more bioactive chemicals into the environment that interact between plants with either stimulatory or inhibitory influences, i.e. a phenomenon known as allelopathy [6] which was first put forward to describe the effect of ethylene on the fruit ripening from physiological perspective [7]. Allelopathy is usually interspecific [8–9], but also may occur within the same species, which is called autotoxicity [10]. In forest ecosystems, many examples of autotoxicity exist in coniferous trees [11–17]. Autotoxicity was a potential functional process that could influence early recruitment including germination and seedling growth with emphasis on the natural regeneration of *Pinus halepensis* [15]. A spruce-specific metabolite named as *p*-hydroxyacetophenone was isolated in spruce through fall and organic layer showed negative effects on root elongation of spruce seedlings [14]. Previous studies suggested autotoxicity could result in inhibition of seedlings growth or delayed germination, limited offspring [18]. Such regulations could reduce the intensity of intraspecific competition and damage the fitness of the dominant members of a population [19]. For these reasons, autotoxicity has been argued as a cause of forest regeneration failure [20].

Allelochemicals were excreted from plants during the processes of secondary metabolism and accumulated in plants, soils and other organisms [21]. In the latter research, a biotic stress was termed as allelochemicals stress [22], where these allelochemicals negatively affect the growth and trigger a series of morphological and physiological variations in the target plants. Production of large number of reaction oxygen species (ROS) by the plants in response to allelochemicals stress has been suggested. In response to ROS, it is proposed that the allelochemicals extracted from cucumbers such as peroxidase (POD) and superoxide dismutase (SOD) can significantly activate antioxidant mechanisms [5]. The effects of methanolic extracts from *Phytolacca latbenia* on the activities of antioxidant enzymes such as POD, SOD and catalase (CAT) in the geminating seeds of *Brassica napus* and *Triticum aestivum* were also checked, indicating that the activities of POD and SOD were significantly decreased, but CAT activity presented a linear increase in both tested seeds with increasing the concentration of allelochemicals [23]. Additionally, plant hormones regulate several aspects of plant growth and development processes in response to the multiple abiotic and biotic stresses [24–27]. The level changes of hormones in plants due to allelochemicals also have been reported. For examples, the methanol extracts of *Lepidium draba* were found to increase the ABA level and significantly decrease GA_3_ level of corn and redroot pigweed [28], and the aqueous leachate of *Sicyos deppei* caused higher content of ABA during all times of tomato germination [29]. However, the physiological mechanism of autotoxicity remains to be elucidated.

Schrenk spruce (*Picea schrenkiana*) as one typical species of boreal forest, is mainly distributed on the northern and southern slopes of Tianshan Mountains and the northern slope of Kunlun Mountain West in China., *P*. *schrenkiana* has been received much attention in the ecological aspect, because it plays an important role in water and soil conservation and to maintain balance of ecosystem. However, the natural regeneration of *P*. *schrenkiana* has been in jeopardy [30]. It has been hypothesized that secondary metabolites released by litter and root secretion were accumulated around the rhizosphere due to fire suppression, which caused a autotoxic effect to the regeneration of *P*. *Schrenkiana*. 3,4-dihydroxy-acetophenone (DHAP) was proved to be the major allelochemical in *P*. *schrenkiana* needles and litter [30–32]. In natural forest condition, 0.51 mg/g DHAP was contained in dry soil of a mature *P*. *schrenkiana* forest. The concentration of DHAP would be 0.224 mM when 0.51 mg DHAP was dissolved into 15 mL snow or rain water [32]. According to the previous studies, DHAP stress was considered to be a biotic stress to *P*. *schrenkiana*. In this work, therefore, series of experiments including root cell viability, antioxidant enzymes activities and plant hormones content were designed and conducted to explore physiological mechanism of *P*. *Schrenkiana* treated with different concentrations of DHAP.

## Materials and methods

### Chemicals, plant material and reagents

DHAP was isolated in our laboratory [31–32]. Seeds were collected from pure stands of *P*. *Schrenkiana* located at the forest farm of Xinjiang Agricultural University (2 198 m, 43°22′58"N, 86°49′33"E) on September, 2014. All Seeds were selected from healthy plants without infection and stored at 4°C. All other chemicals and solvents in analytical grade were purchased from commercial sources.

### DHAP treatment on seed germination

100 grains of seeds were preceded in plastic boxes (12×12 cm) lined with two layers of filter paper in five replicates, and 10 mL DHAP at concentrations of 0, 0.1, 0.25, 0.5 and 1.0 mM were added into each box, respectively. Seeds were incubated in an artificial intelligence simulation incubator under a 16/8 h (day/night) photo period with photon flux density of 40 μmol·m^-2^s^-1^ at a day/night temperature of 12/4°C. When radicle emerged, the seeds were considered germination after incubation. Germination rate was calculated after 15 days and germination vigor was calculated on the tenth day of DHAP treatment. The under-germinated seeds at 3 days were selected to determine the antioxidant enzymes activities and the levels of plant endogenous hormones.

### DHAP treatment on seedlings growth

*P*. *Schrenkiana* seeds were pre-germinated in plastic boxes lined with filter paper until radicle emergence. Subsequently, 100 grains of successful germination seeds were placed in Petri dishes in five replicates, and 10 mL DHAP (0, 0.1, 0.25, 0.5 and 1.0 mM) was added into each dish, respectively. The cultural conditions of seedlings growth were under a 16/8 h (day/night) photo period with photon flux density of 40 μmol·m^-2^s^-1^ at a day/night temperature of 14/6°C. The length of radicle of five seeds randomly sampled from each Petri dish, was measured with a vernier caliper (GB/T 1214.2–1996, Measuring Instrument LTD, Shanghai). Meanwhile fresh weight of *P*. *Schrenkiana* seedlings was also recorded. After five days, these parameters were taken measurements, and continued once every five days for a total of 20 days. The seedlings after the measurement of radicle length and fresh weight were used to determine the antioxidant enzymes activities and the levels of plant endogenous hormones.

### Root cell viability of seedling

The viability of *P*. *Schrenkiana* root cell was referred to the method of double staining with fluorescein diacetate (FDA) and propidium iodide (PI) [33]. Root tissues (0.1–1 cm length from the tip) were excised from the intact *P*. *Schrenkiana* seedlings treated with 0, 0.1, 0.25, 0.5 and 1.0mM DHAP. The root tissues were stained with a mixture of 12.5 μg·mL^-1^ FDA and 5 μg·mL^-1^ PI for 10 min at room temperature in the dark and then washed with distilled H_2_O. The slit root tissues were observed and photographed using a fluorescence microscope (Nikon E600 with a B-2A filter, excitation 450–490 nm, emission at 520 nm, Nikon Corp., Tokyo, Japan).

Evans blue staining was the other method to evaluate cell viability [34]. The intact *P*. *Schrenkiana* seedlings were treated with different concentration of DHAP for 3, 6 and 9 days, respectively. After the roots were washed with distilled H_2_O, several seedlings roots (0.1–1 cm from the tip) were stained in 0.25% (w/v) aqueous solution of Evans blue for 1 h at 30°C in the dark. Thereafter, the stained roots were washed with distilled H_2_O for 10 min and then extracted with N,N-dimethylformamide without grinding for 24 h at 30°C in the dark. Finally absorbance of the released Evans blue was measured using spectrophotometer (Beckman DUO 640; Beckman Coulter Inc., Fullerton, CA, USA) at 600 nm.

### Assay of antioxidant enzyme activities in *P*. *Schrenkiana*

Tissues (0.1 g) were weighed and ground in 1 mL phosphate buffer (50 mM, pH 7.8) containing 1 mM EDTA and 2% (w/v) polyvinyl pyrrolidone (PVP) using chilled mortar and pestle. The homogenate was filtered for two times and centrifuged at 10,000 r·min^-1^ for 20 min at 4°C, and the clear supernatant was then used to determine the antioxidant enzyme activities except APX activity. For measuring APX activity, the tissue was homogenized in phosphate buffer (50 mM, pH 7.8) supplemented with 2 mM ascorbate, 1 mM EDTA and 2% (w/v) PVP. The parallel control was run where distilled H_2_O was used instead of enzyme extract. All spectrophotometric analyses were conducted at 25°C in a Shimadzu UV/Visible Light spectrophotometer.

Superoxide dismutase (SOD) activity was determined based on the inhibition of the photochemical reduction of nitroblue tetrazolium (NBT) according to Giannopolitis and Ries [35]. The reaction mixture (6.6 mL) consisted of 50 mM phosphate buffer (pH 7.8) 3 mL, 130 mM methionine 0.6 mL, 750 μM NBT 0.6 mL, 20 μM riboflavin 0.6 mL, enzyme extract 0.2 mL, 0.1 mM EDTA 0.6 mL and distilled H_2_O 1 mL. The reaction was conducted at 25°C under 4,000 lx for 15 min. After illumination, absorbance of solution was measured at 560 nm. One unit of SOD activity was defined as that amount of enzyme that caused 50% inhibition of NBT reduction.

Peroxidase (POD) activity was detected by guaiacol method [36]. The reaction mixture was 4 mL including 0.1 mL enzyme extract, 1.9 mL phosphate buffer at 50 mM, 1 mL guaiacol solution at 50 mM and 1 mL 2% H_2_O_2_. The increase in the absorbance was measured at 470 nm as guaiacol oxidation recorded at 30 s intervals up-to 2 min. One unit of POD activity was defined as the amount of enzyme increased 0.01 in the absorbance at 470 nm per min [37].

Catalase (CAT) activity was measured from the rate of H_2_O_2_ decomposition as measured by the decrease of absorbance at 240 nm, following the procedure of Lee *et al* [38]. The reaction mixture (3 mL) contained 100 μL enzyme extract and 2.9 mL phosphate buffer (10 mM H_2_O_2_ included) at 50 mM. One unit of CAT activity was calculated as the amount of enzyme reduced 0.01 in absorbance at 240 nm per min [37].

Ascorbate peroxidase (APX) activity was determined according to Nakano and Asada [39]. The reaction mixture (3 mL) was composed of 2.5 mL phosphate buffer (containing 0.5 mM ascorbate) at 50 mM, 50 μL H_2_O_2_ at 6 mM and 450 μL enzyme extract. The hydrogen peroxide-dependent oxidation of ascorbate was followed by a decrease in the absorbance at 290 nm. APX activity was expressed as 1 mM ascorbate oxidized per min.

The guaiacol peroxidase (GPX) was determined by the modified method described by Cakmak and Marschner [40]. The reaction including 1 mL phosphate buffer at 50 mM, 400 μL guaiacol (containing 2.5 mM NaN_3_) at 1 mM, 200 μL H_2_O_2_ at 1.5 mM and 400 μL enzyme extract was carried out at 37°C for 5 min. The absorbance of 412 nm was recorded. One unit of GPX was defined as the amount of glutathione increased 1 in the absorbance at 470 nm per min.

Glutathione reductase (GR) activity was usually assayed by following GSSG-dependent oxidation of NADPH [41]. The reaction mixture (3 mL) contained 450 μL enzyme extract, 2.34 mL phosphate buffer at 50 mM, 60 μL NADPH at 10 mM and 150 μL GSSG at 10 mM. The decrease in absorbance at 340 nm was monitored for 2 min. One unit of GR activity was expressed as 1 μM NADPH oxidized per min.

### Assay of phytohormones levels in *P*. *Schrenkiana*

Samples (0.1 g) were frozen in liquid nitrogen and instantly ground to a powder. 200 μL cold methanol of 80% (containing 1 mM BHT as an antioxidant) was sequentially added, and the homogenate was temporarily incubated at 4°C in the dark for 12 h. After that, the homogenate was centrifuged at 10 000 r·min^-1^ for 20 min at 4°C. The supernatants were passed through Chromosep C18 columns, prewashed with 80% methanol. The hormone fractions were dried under N_2_, dissolved in 2 mL mobile phase and filtered by 0.22 μm membrane for analysis. Chromatographic analysis was performed using the Agilent 1290 UPLC (Ultra-high Performance Liquid Chromatography) system with a C18 reversed-phase column (Eclipse Plus C18, 2.1×150 mm, 1.8 μm) (Agilent, Santa Clara, CA, USA) set at 30°C. A diode array detector was monitored at 254 nm. Elution with solvent A (methanol/acetonitrile, 5:95) and solvent B (water/acetonitrile, 5:95) in a step gradient manner at a flow rate of 0.5 mL∙min^-1^ was carried out as follows: 0–1 min, 25% A; 1–4 min, 25%–45% A; 4–8min, 45% A; the sample injection volume was 0.3 μL. Phytohormone concentrations (μg∙g^-1^ fresh weight) were automatically calculated from peak area by software using authentic standards run with the samples.

### Statistical analyses

All results were presented as the mean ± standard error of five replications. All data were statistically analyzed using SPSS software (IBM, New York, USA). For statistical analyses, relationships were considered to be significant when *p*<0.05. If the results of One-way ANOVA showed the significant differences at the 0.05 significance level, we used LSD (Least Significance Difference) for multiple comparisons among the different treatments.

## Results

### Effects of DHAP on seed germination

**Fig 1:**
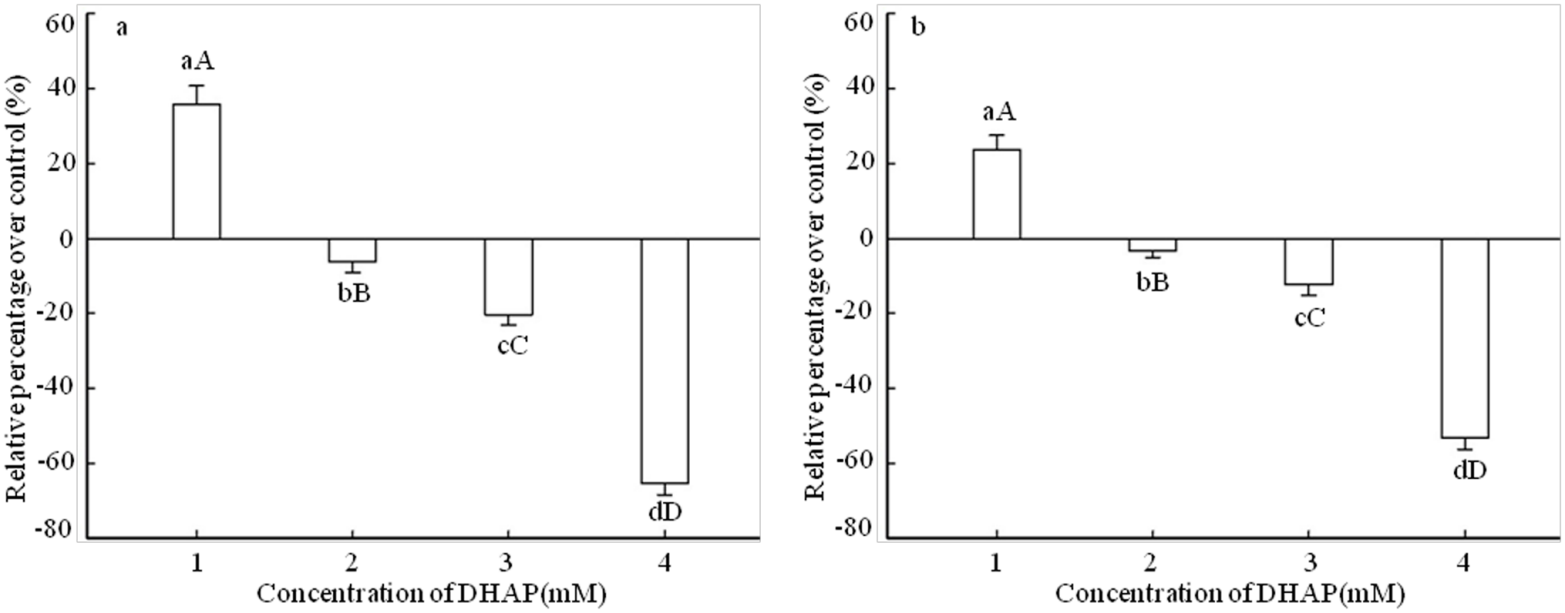
Effect of DHAP on seed germination of *P*. *schrenkiana*. a: Germination rate. b: Germination vigor. 1: 0.1 mM. 2: 0.25 mM. 3: 0.5 mM. 4: 1.0 mM. *Error bars* present standard errors of five independent biological replicates. The same upper- and lower-case letter indicates a non-significant difference (*p* > 0.05), different lower-case letters indicate a significant difference (*p*<0.05), different upper-case letters indicate a strongly significant difference (*p*<0.01).

Effects of DHAP on the germination of *P*. *Schrenkiana* seed were measured by germination rate and germination vigor (Fig 1). In comparison with the distilled water as control, DHAP at 0.1 mM level had a stimulatory effect on seed germination, and the germination rate and vigor remarkably increased by 36% and 24%, respectively. At DHAP concentrations ranged from 0.25 to 1.0 mM, the germination rate and vigor were inhibited, especially at 1.0 mM DHAP level, the germination rate and germination vigor significantly decreased by 65% and 53% respectively, indicating a strong inhibitory effect.

### Effects of DHAP on enzyme activities and phytohormones levels in seed germination

The seeds of P. Schrenkiana were treated with different concentrations of DHAP, and the antioxidant enzymes activities and endogenous hormones levels of seeds were determined after 3 days treatment. The data in Table 1 showed that after treated by 0.5 mM DHAP, the activities of antioxidant enzymes SOD and CAT significantly increased by 58% and 65% higher than the control respectively, but the treatment of DHAP at high concentration (1.0 mM) reduced SOD and CAT activity by 9% and 19%, compared with the control. Unlike SOD and CAT, the activities of antioxidant enzymes POD, GPX and GR tended to be stimulated as DHAP concentration increased, such as the significant increase of 56% in POD activity by the treatment of DHAP at 1.0 mM, the obvious increases of 42% and 20% for GPX and GR activities at the concentration of 0.5 mM. However, the activity of APX enzyme was increased at low DHAP concentration (0.1–0.25 mM), but reduced at high DHAP concentration (0.5–1.0 mM). In general, the activities of all the antioxidant enzymes except for APX were higher than the control at 0.5 mM DHAP concentration, indicating that *P*. *Schrenkiana* can make a positive self-protection effect at moderated DHAP concentration, but a negative self-inactivation effect at high DHAP concentration of 1.0 mM due to the decay of antioxidant enzymes activities, which in turn affected the seeds germination.

**Table 1:** Effects of DHAP on the antioxidant enzymes activities and phytohormones content during the seed germination.

Physiological index	Parameters	DHAP concentration (mΜ)				
		0	0.1	0.25	0.5	1.0
**Antioxidant enzymes activities**	SOD(U∙g^-1^)	34.18±2.02cB	37.54±4.61bcB	42.21±5.47bB	53.81±1.37aA	31.20±3.84cB
	POD(U∙g^-1^)	29.34±1.93bcB	29.07±1.44bcB	26.89±4.49cB	34.40±2.42bB	45.62±5.71aA
	CAT(U∙g^-1^)	22.63±2.60bcB	24.87±1.93bcB	28.72±3.01bAB	37.40±3.49aA	18.22±2.21cB
	APX(μΜ ASA ∙g^-1^)	835.02±31.02cC	897.00±51.42bB	1124.52±102.47aA	828.91±71.48cC	741.63±39.73dD
	GPX(U∙g^-1^)	825.41±26.07eE	869.05±46.10dD	902.12±31.74bB	1169.17±98.12aA	947.83±100.20cC
	GR(μΜ NADPH ∙g^-1^)	19.72±2.93aA	20.4±1.06aA	20.2±2.45aA	23.7±3.01aA	21.7±2.12aA
**Phytohormones content**	IAA(μg∙g^-1^)	14.8±0.32bB	12.6±0.43cB	18.4±1.41aA	12.2±1.12cB	8.7±1.10dC
	GA_3_(μg∙g^-1^)	10.32±1.59bBC	11.29±0.83bB	14.37±0.75aA	9.24±0.92cC	7.85±0.54dC
	ZT(μg∙g^-1^)	1.50±0.10aA	1.58±0.07aA	1.72±0.25aA	1.28±.0.16aA	0.94±0.05aA
	ABA(μg∙g^-1^)	0.18±0.02cC	0.14±0.04dCD	0.11±0.03dD	0.24±0.04bB	0.37±0.05aA

Note: The same upper- and lower-case letter indicates a non-significant difference (*p* > 0.05), different lower-case letters indicate a significant difference (*p*<0.05), different upper-case letters indicate a strongly significant difference (*p*<0.01).

To analyze the changes in endogenous hormones of *P*. *Schrenkiana seeds*, chromatogram of ZT, GA_3_, IAA, ABA and DHAP by UPLC was conducted and showed that calibration curves of ZT, GA_3_, IAA and ABA were linear and the *R*^*2*^ values were in the range 0.9995–0.9998, presenting good linearity. The UPLC analyses of the plant hormones suggested that DHAP at moderate concentration significantly increased the levels of ZT, GA_3_ and IAA, but at high concentration inhibited the levels. The highest levels of ZT, GA_3_ and IAA were observed at 0.25 mM DHAP, increased by 15%, 39% and 24% in comparison with the control, respectively, and the lowest levels of ZT, GA_3_ and IAA were found in the treatment of 1.0 mM DHAP, reduced by 37%, 24% and 41%, respectively (Table 1). As an exception, the level of hormone ABA was decreased at low DHAP concentration, especially at 0.25 mM DHAP (39% decrease), while the high DHAP concentration had a significantly stimulatory effect on the ABA level. Furthermore, a remarkable elevation about 106% was found at 1.0 mM DHAP concentration (Table 1), indicating that the accumulation of ABA level was the main cause to delay the germination of *P*. *Schrenkiana* seeds, as well known that ABA delays or inhibits seed germination.

### Effect of DHAP on seedling growth

**Fig 2:**
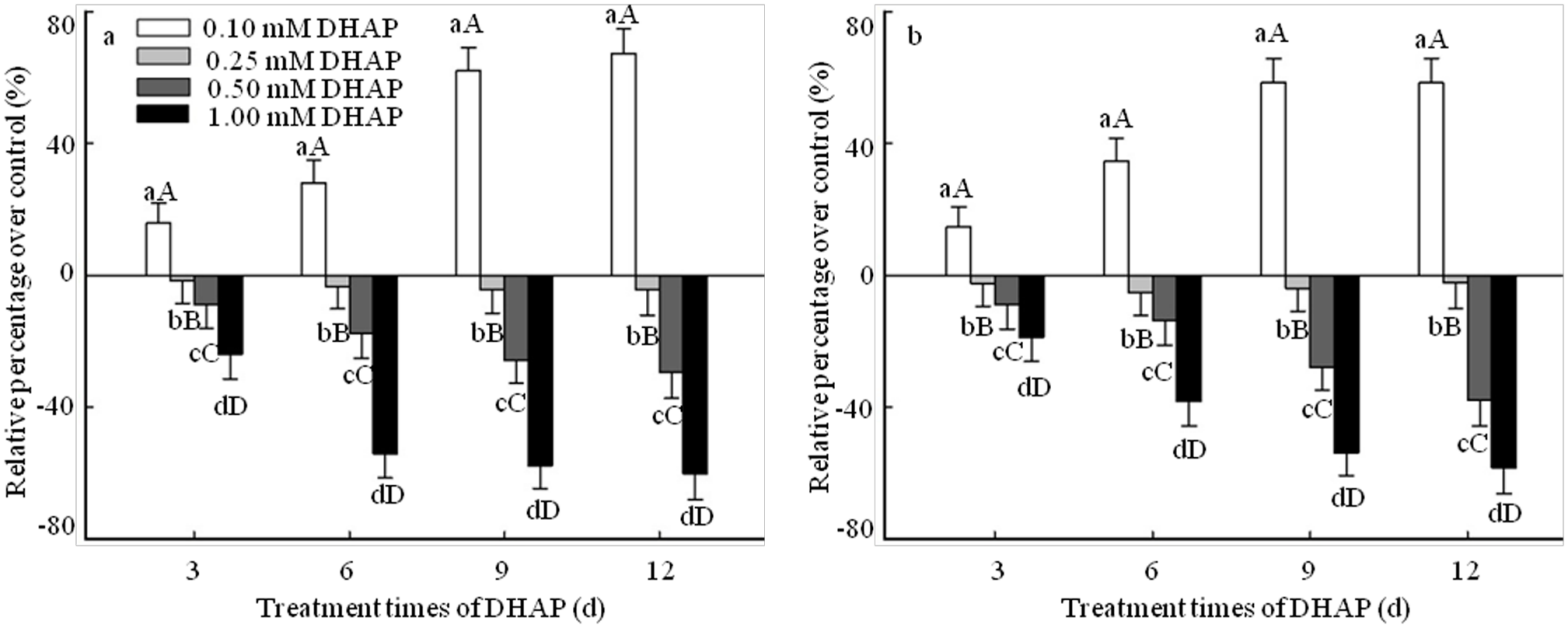
Effect of DHAP on seedlings growth of *P*. *schrenkiana*. a: Radicle elongation. b: Fresh weight. *Error bars* present standard errors of five independent biological replicates. The same upper- and lower-case letter indicates a non-significant difference (*p* > 0.05), different lower-case letters indicate a significant difference (*p*<0.05), different upper-case letters indicate a strongly significant difference (*p*<0.01).

Radicle length and fresh weight are often defined as seedling growth parameters, and thus they were adopted to determine the effect of DHAP on seedling growth of *P*. *Schrenkiana* (Fig 2). In general, DHAP at low concentration (0.1 mM) had a significant hormesis on both the radicle length and fresh weight in the early stage of seedlings growth. Compared with that of the control, the increase of the radicle length and fresh weight were reached the maxima of 67% and 58% respectively at 12 days of DHAP stress treatment. However, it showed inhibitory effect on the development of seedlings in particular that the DHPA at 1.0 mM decreased the radicle length and fresh weight by 60% and 58% in comparison with the control after 12 days treatment, respectively (Fig 2). These results implied that DHAP at high concentration (≥0.25 mM) had inhibitory effect on the development of *P*. *Schrenkiana* seedlings, but it gave stimulatory effect at low concentration (0.1 mM).

### Effect of DHAP on cell viability in seedling roots

**Fig 3:**
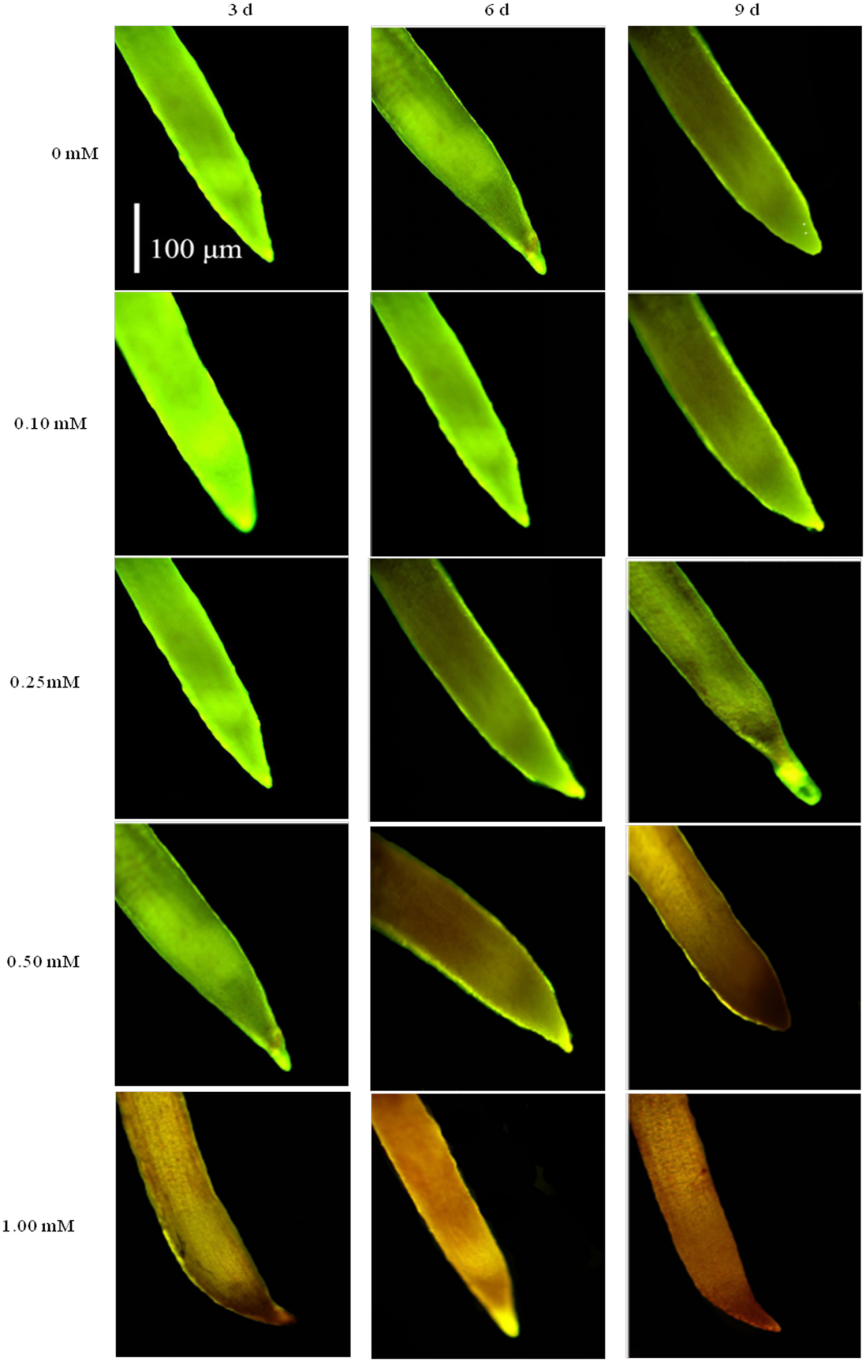
Effects of DHAP on root tips viability of *P*. *schrenkiana* seedlings tested by FDA-PI staining.

The cell viability in *P*. *Schrenkiana* root was determined by a double-staining method using FDA-PI (Fig 3). The double-staining analysis demonstrated that the root tip treated by DHAP at 0.1 mM presented green fluorescence after 9 days, indicating that cells were viable. The root tip treated with 0.25 mM DHAP revealed green fluorescence after 6 days and reddish brown fluorescence after 9 days, respectively. But a reddish brown fluorescence in the 0.5 mM DHAP-treated root tip after 6 days clearly indicated the death of cells. Compared with control treat, DHAP at 1.0 mM level induced cell death after 3 days treatment. Therefore, the damage of 0.1 and 0.25 mM DHAP on root tip cell was less than that of 1.0 mM DHAP, which was more serious.

For further study, Evans blue staining quantified the rates of cell death was also adapted to determine cell viability in *P*. *Schrenkiana* roots (Table 2). DHAP at 0.5 and 1.0 mM enhanced significantly Evans blue uptake of the roots after 3, 6 and 9 days treatment compared with control, while the uptake level of the roots treated by 0.1 mM DHAP after 3 and 6 days was much lower than that of the control. The uptake of Evans in the root cells treated by 0.25 mM was presented a slight increase compared with control. This indicates that DHAP at 1.0 mM concentration has higher activity to induce the death of the root cells than low concentration DHPA. In addition, the results of Evans blue staining coincided with the results of FDA-PI staining on root cells.

**Table 2:** Effect of DHAP on Evans blue uptake on the roots of *P*. *schrenkiana* seedlings.

DHAP (mM)	Relative Evans blue uptake (%)		
	3 d	6 d	9 d
**0**	100.00±15.34cC	107.66±14.37cC	114.65±18.08 cC
**0.1**	86.77±14.23dD	92.76±23.86dD	114.56±26.69cC
**0.25**	98.65±10.470cC	102.56±21.72cC	123.59±22.72cC
**0.5**	150.00±25.47bB	220.57±25.42bB	280.45±14.89bB
**1.0**	276.34±26.53aA	302.59±20.17aA	305.62±19.37aA

Note: The same upper- and lower-case letter indicates a non-significant difference (*p* > 0.05), different lower-case letters indicate a significant difference (*p*<0.05), different upper-case letters indicate a strongly significant difference (*p*<0.01).

### Effect of DHAP on enzymes activities of seedlings

**Fig 4:**
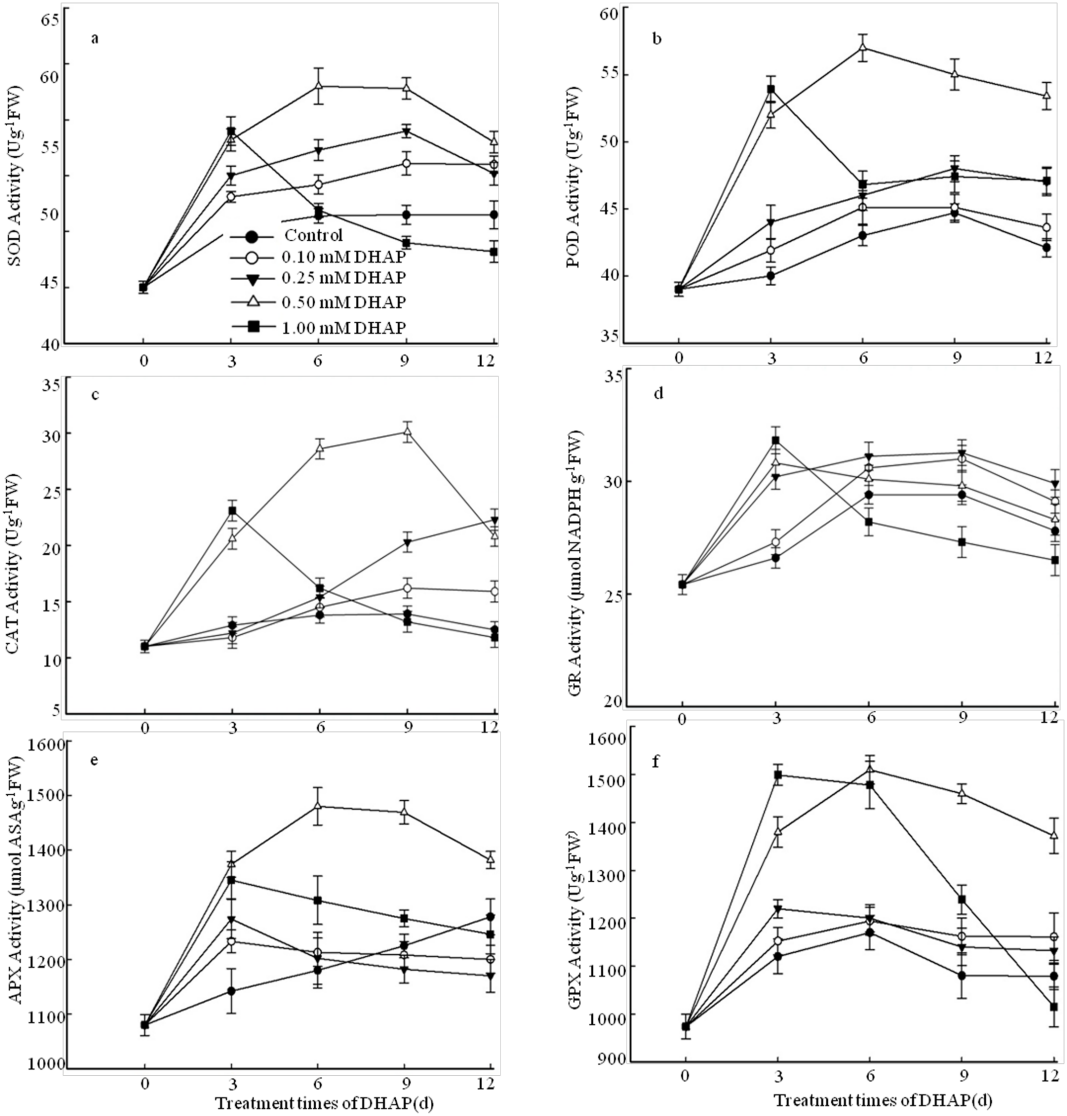
Effects of DHAP on the antioxidant enzymes activities during the early seedlings growth. a: SOD. b: POD. c: CAT. d: GR. e: APX. f: GPX. *Error bars* present standard errors of five independent biological replicates.

Activities of enzymes SOD and POD were monitored at 3, 6, 9 and 12 days of DHAP stress in *P*. *Schrenkiana* seedlings (Fig 4a and 4b). SOD activity of *P*. *Schrenkiana* seedlings remained unchanged in comparison with the control. As the degree of DHAP stress increased, the SOD activity increased initially during early days of growth except for the 1.0 mM DHAP concentration, and reached a maximum at 6 days (30% increase) under 0.5 mM DHAP treatment (Fig 4a). Compared with the control, SOD activity of *P*. *Schrenkiana* seedlings treated with 1.0 mM DHAP increased initially and then declined. Like the SOD activity, a significant decrease about 24% was also observed in POD activity after 6 grown days under 0.5 mM DHAP treatment and the enzyme activity was decreased under 1.0 mM DHAP (Fig 4b). The activity of CAT increased in *P*. *Schrenkiana* seedling treated with DHAP during early days of growth and reached the maximum at 9 days under 0.5 mM DHAP treatment, but decreased thereafter under 1.0 mM DHAP level (Fig 4c). By treated with 0.5 mM DHAP, an obvious increase about 1.1 times was observed in enzyme activity at 9 days as compared to the control plants. CAT activity of *P*. *Schrenkiana* seedlings treated with 1.0 mM DHAP declined, whereas a transient increase was found at 3 days treatment. The activities of SOD, POD and CAT were all increased under 0.5 mM DHAP treatment, indicating that moderate DHAP stress can increase the resistance ability of *P*. *Schrenkiana*, but 1.0 mM DHAP inhibited the activities of these enzymes, due to the reduction of tolerance to high DHAP stress. APX and GPX play important roles in the H_2_O_2_ scavenging system, thus we examined the APX and GPX activities in *P*. *Schrenkiana* seedling subjected to DHAP stress during early grown days (Fig 4e and 4f). The APX activity of *P*. *Schrenkiana* seedling induced by 0.1 and 0.25 mM DHAP had a transient increase at 3 days treatment and then decreased with extending the days, as compared to the control. Unlike APX activity variation, GPX activity was slightly increased for early grown 12 days. A significant increase in APX and GPX activity was detected under high DHAP (0.5 and 1.0 mM) after 3 days and reached maximum after 6 days under 0.5 mM DHAP stress, and then decreased thereafter. The obvious elevation of APX and GPX activities about 25% and 29% was observed as compared to the control plants. Unlike other antioxidant enzymes, the activity of GR activity had little changes in the presence or absence of DHAP (Fig 4d).

### Effect of DHAP on phytohormones level in seedlings

**Fig 5:**
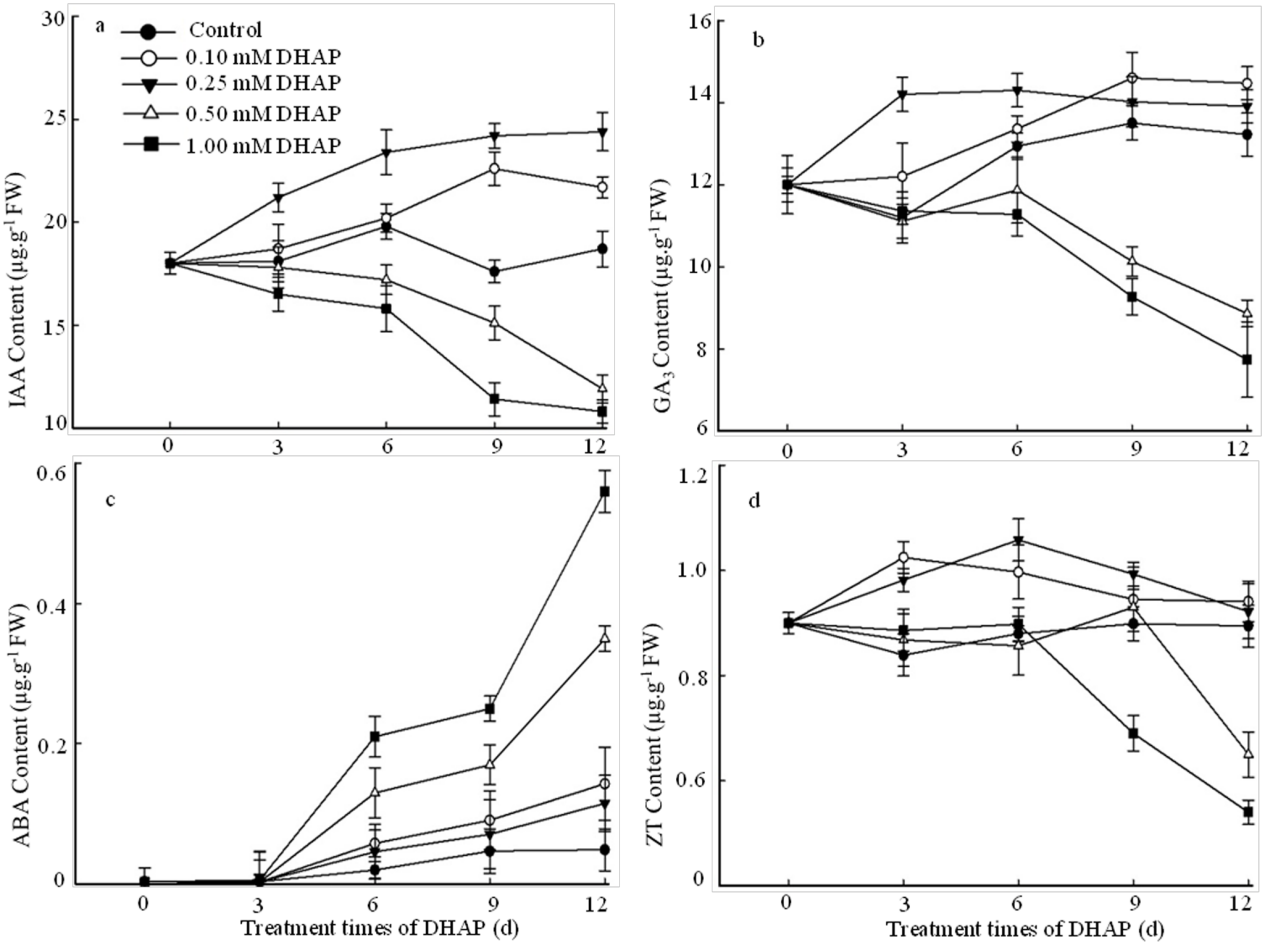
Effects of DHAP on the content of endogenous plant hormones during the early seedlings growth. a: IAA; b: GA3; c: ABA; d: ZT. *Error bars* present standard errors of five independent biological replicates.

The endogenous ZT, GA_3_, IAA and ABA levels of *P*. *Schrenkiana* treated with different concentration of DHAP were compared in Fig 5. A time-course study revealed that the levels of phytohormones induced by DHAP increased under the low concentration during early 12 days of grown, but the levels were inhibited significantly under high DHAP concentration. An obvious elevation about 40% and 28% in IAA level was observed in 9 days grown seedlings at 0.25 and 0.1 mM DHAP levels, respectively. The levels of GA_3_ and ZT increased by 26% and 22% respectively in 3 grown days of seedlings at 0.25 mM DHAP. After 12 days exposed to 1.0 mM DHAP, the seedlings exhibited significant decreases of 64%, 42% and 50% in IAA, GA_3_ and ZT levels. Moreover, the seedlings exposed to 0.5 mM DHAP exhibited the decreases of 52%, 34% and 28% in comparison with those seedlings in absence of DHAP after 12 days. Unlike the levels of IAA, GA_3_ and ZT, an increase of ABA level was found along with the DHAP treated times. In addition, the seedlings induced by the treatment of 1.0 mM DHAP had a significantly higher ABA level than those treated with the control, 0.1 mM and 0.25 mM DHAP (13.3, 3.8, and 2.0 folds), respectively (*p*<0.01).

## Discussion

Some compounds have been found to exhibited concentration-dependent stimulatory or inhibitory effects on seedling growth [42–44]. 4, 8-Dihydroxy-1-tetralone (4,8-DHT) isolated from *Carya cathayensis* had a hormesis at low concentration, but significantly inhibited seedling growth of lettuce at the high concentration, [44]. Needle-leached DHAP had a similar effect on some of the plants. DHAP promoted the seeds germination and seedlings growth of *P*. *Schrenkiana* at low concentration (≤ 0.1 mM), but had a significant inhibition at high concentration (≥0.5 mM). In this investigation, 0.25 mM DHAP presented a slight promotion and inhibition on seeds germination and seedlings growth of *P*. *Schrenkiana*, so it was hypothesized that 0.25 mM DHAP might be the inflection point of changing action direction. It was near to the concentration of 0.224 mM in natural forest conditions [32]. Thus, our investigation provided evidence on the phytotoxic potential of *P*. *Schrenkiana*.

FDA-PI is a rapid, convenient, reliable and simultaneous double-staining procedure to determine the cell viability [45–47]. To study the effect of DHAP on the viability of *P*. *Schrenkiana* root cell, a double staining experiment was conducted according to this procedure. FDA readily enters intact cells and undergoes hydrolysis by endogenous esterase to releases free fluorescence [48]. PI readily enters the cells with injured membranes and can be detected by its red fluorescence [48]. Therefore, it is used to detect dead cells [49]. In this study, high concentration of DHAP induced cell death in root tip cells after 3 days treatment. Similar findings were reported in the studies on phytotoxic activities of _L_-DOPA exuded from *Mucuna spp*., which induced cell death [49].

Allelochemicals as biotic stress exhibit a wide range of action mechanism, from effects on DNA, photosynthetic, ion uptake water balance, and the activities of antioxidant enzymes and plant hormones [5, 28, 50–52]. SOD, POD, CAT, APX and GPX are the major antioxidant enzymes [23, 53–55], and GR plays an important role in maintaining a high GSH/GSSG ratio in plants [56]. Within a cell, the SOD is considered the first line of defense against the ROS, as well as a key antioxidant enzyme to convert O_2_^−^ into H_2_O_2_ and O_2_ [57]. Subsequently, both CAT and APX are correlated to consume H_2_O_2_ [58]. In the present study, DHAP had different effects on antioxidant enzymes activities in *P*. *Schrenkiana* seedlings, respectively. The activities of all antioxidant enzymes except for GR were significantly increased at DHAP concentration of 0.5 mM during the early days of seedlings growth. It can be concluded that the moderate concentration of DHAP can increased the activities of antioxidant enzymes to help *P*. *Schrenkiana* seedlings maintain the ROS levels well below to their deleterious levels to enhance the resistance of *P*. *Schrenkiana*. These results agree with other studies described antioxidant enzymes under allelochemical stress. It has been reported that low and medium ginsenoside isolated from ginseng, significantly stimulated the activities of SOD, POD and CAT of treated roots of American ginseng [59]. Likewise ferulic acid increased antioxidant enzymes in maize seedlings [60], and benzoic acid in cucumber cotyledons [61]. That is a self-protective mechanism of plants in response to biotic and abiotic stresses. But the activities of the antioxidant enzymes were decreased at 1.0 mM toxicity level. A reduction in enzymes activities has also been observed in other studies on allelochamical modes of actions, that is, two allelochemicals isolated from the leachates of *Ageratina adenophora* decreased POD and SOD activities in rice seedlings under high concentration after 48 h treatment [52]. It is speculated that the accumulation of ROS induced during severe DHAP stress goes beyond the clearance ability of antioxidant enzymes. Excessive ROS can induce cell damage which in turn can induce *P*. *Schrenkiana* seedlings death. In addition, previous researches have shown that various allelochemicals could change plant hormone levels of crops and weed [28, 62–63]. Evidences from physiological studies indicated that IAA, ZT and GA_3_ affected cell enlargement and balanced the plant growth [64–66]. The present studies showed that different concentrations of DHAP affected the level of IAA, ZT and GA_3_. It was probable that low concentration of DHAP increased the levels of IAA, ZT and GA to promote the growth of seedlings, while high concentration of DHAP inhibited the level of IAA, ZT and GA_3_ and subsequently blocked extension growth. The radicle length and fresh weigh were related to the content of IAA, ZT and GA_3_ affected by DHAP. This was parallel to the results by treated with other abiotic and biotic stresses [67–69]. On the other hand, the level of ABA was significantly higher in the DHAP-treated seedlings than that of the control, indicating that the elevated DHAP stress increased the ABA content, which was an adaptation process in response to DHAP stress. These results suggested that the endogenous hormones might have interactive effects on *P*. *Schrenkiana* seedlings to respond and adapt the DHAP stress. Thus, a further study is thereby needed to determine how endogenous hormones regulate the growth of *P*. *Schrenkiana* seedlings under DHAP stress.

## Conclusion

The present investigation suggested that DHAP as allelochemical is one of the many possible factors contributing to the failure of *P*. *Schrenkiana* natural regeneration. According to the experiment, the DHAP isolated from *P*. *Schrenkiana* was found to inhibit germination, radicle elongation and fresh weight of *P*. *Schrenkiana* at high concentration and had a hormesis at low concentration. DHAP had different effects on antioxidant enzymes activities and plant hormones levels in *P*. *Schrenkiana* seedlings, respectively. The moderate concentration of DHAP increased antioxidant enzymes activities, favorable to disturb the balance between production and scavenging of ROS, and in turn excessive ROS induced by high DHAP concentration could inhibit the *P*. *Schrenkiana* seedlings growth. Moreover, DHAP induced significant cellular damages, which played a major role in inhibition of radicle elongation and tolerance to DHAP. In present study, all experiments were carried out in the simulated environment. More research is needed for further evaluation using pot and field experiments for better understanding the autoxicity potential of *P*. *Schrenkiana* under field conditions. Besides, other possible factors involved in the natural regeneration of *P*. *Schrenkiana* such as stand structure and the deterioration of soil physicochemical properties are needed for further evaluation.

## Supporting information

S1 File Supporting information was the raw data underlying the findings of a paper. (XLSX)
